# Reply to comment by Compagnoni *et al.* on “First finding of continental deep subduction in the Sesia Zone of Western Alps and implications for subduction dynamics”

**DOI:** 10.1093/nsr/nwae457

**Published:** 2024-12-12

**Authors:** Yi-Xiang Chen, Kun Zhou, Qiang He, Yong-Fei Zheng, Hans-Peter Schertl, Kun Chen

**Affiliations:** State Key Laboratory of Lithospheric and Environmental Co-evolution, University of Science and Technology of China, China; Center of Excellence for Comparative Planetology, Chinese Academy of Sciences, China; State Key Laboratory of Lithospheric and Environmental Co-evolution, University of Science and Technology of China, China; State Key Laboratory of Lithospheric and Environmental Co-evolution, University of Science and Technology of China, China; State Key Laboratory of Lithospheric and Environmental Co-evolution, University of Science and Technology of China, China; Center of Excellence for Comparative Planetology, Chinese Academy of Sciences, China; Institute of Geology, Mineralogy and Geophysics, Faculty of Geosciences, Ruhr University Bochum, Germany; State Key Laboratory of Lithospheric and Environmental Co-evolution, University of Science and Technology of China, China

The occurrence of coesite in metamorphic rocks of supracrustal origin demonstrates that the low-density continental crust was subducted to subarc depths for ultrahigh-pressure (UHP) metamorphism [1,2]. Thus, the finding of coesite in supracrustal rocks is critical to answer a series of pivotal questions concerning continental deep subduction, regional metamorphism, the tectonic evolution of microplate margins and the recycling of crustal materials in continental collision zones. In this regard, we welcome the comment by Compagnoni and his colleagues on our work [3], which provides us an opportunity to clarify some ambiguities, highlight our key results and propose the directions for future work.

The critiques of Compagnoni *et al.* [4] are mainly on the unusual occurrence of coesite and its Raman spectroscopy evidence presented in our paper [3]. The first concern is that they envisage the possibility of contamination by dirt or polishing material based on the observation that the analytical spots were at the contact between the exposed quartz inclusions and the host garnet. However, neither known polishing materials (like Al_2_O_3_, Cr_2_O_3_, MgO, SiC powder, or diamond powder) nor dirt are characterized by a Raman spectroscopic peak at 521 cm^−1^. Although monocrystalline silicon has a Raman shift at ∼520 cm^−1^, it cannot be contaminated into the thin section that is made of glass. No monocrystalline silicon was involved in the production of our thin sections. This scenario is distinct from the identification of diamond in the metamorphic rocks of supracrustal origin, for which the diamond powder used in polishing of thin sections is indeed a big problem.

**Figure 1.**
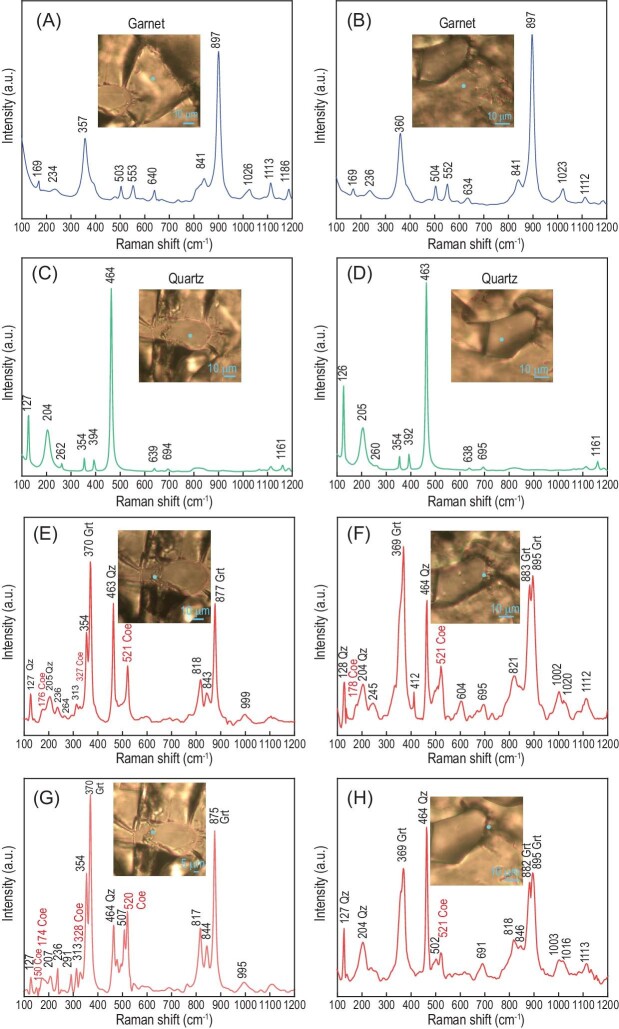
The representative Raman spectra for individual mineral garnet (A and B), quartz (C and D) and the coesite-bearing inclusions in garnet (E–H) with Raman shift at 100–1200 cm^−1^. Mineral abbreviations: Grt, garnet; Qz, quartz; Coe, coesite.

The second concern is the possible overlapping of peaks, which may influence the identification of Raman spectroscopic peak at 521 cm^−1^ for coesite. Compagnoni *et al.* [4] proposed that garnet has an A_1__g_ mode between 515 and 570 cm^−1^, which may correspond to the 521 cm^−1^. Our studied garnet is mainly the solid solution of almandine and grossular (Alm_37.2–49.0_Grs_43.6–53.4_Sps_5.2–8.9_; Table S6 in Chen *et al.* [3]). Although the garnet of grossular, uvarovite and andradite composition could have the A_1__g_ mode between 516 and 526 cm^−1^ as invoked by Compagnoni *et al.* [4], the peaks are absent for the garnet with a composition of almandine [5]. To test the possible influence of garnet on the Raman shift at ∼521 cm^−1^, we have plotted the Raman spectral data of the studied garnet in Fig. 1. It clearly shows that the analytical spot on individual garnet does not contain the peak at 521 cm^−1^ (Fig. 1A–B), refuting the possibility that the peak of 521 cm^−1^ is influenced by garnet.

The third concern is the interpretation of Raman spectra. The hypothesis of Compagnoni *et al.* [4] is that the relative Raman shift intensity for each mineral in the composite spectra reflecting the overlapping of several minerals should the same as that of individual minerals. To test this hypothesis, we have plotted the composite spectra and compared them with that of individual minerals (Fig. 1). It is known that garnet has two groups of peaks at 876–916 cm^−1^ and 342–376 cm^−1^ that are most prominent, and the range of Raman shifts is dependent on the garnet composition [5]. For our sample, the analytical spot on the individual garnet shows the strongest intensity at ∼897 cm^−1^, following peaks at 357–360 cm^−1^ and 552 cm^−1^ (Fig. 1A–B). However, for the composite spectra, peaks at 875–895 cm^−1^ have weaker intensity than that at ∼370 cm^−1^, and a peak at ∼552 cm^−1^ is nearly invisible (Fig. 1E–H). Although the detailed reason that causes the change of relative Raman shift intensity is unclear, such observations indicate that it is unreliable to assign the Raman peak based on the relative intensities obtained in individual minerals (or sometimes called the standard mineral for comparison of Raman spectra).

Furthermore, the minor peaks of one mineral can also be absent or difficult to be clearly identified in the composite spectra. Based on the relative intensity of Raman shift for coesite, Compagnoni *et al.* [4] proposed that the peak at 327 cm^−1^ assigned to coesite is weaker than expected. They suggested that this peak can be also caused by the overlapping of garnet, especially for the garnet of andradite composition. As argued above, the relative intensity in composite spectra is not a reliable indicator for identification of minerals. Although the garnet of andradite composition could indeed show the peak at 325 cm^−1^ [5], our studied garnet is mainly the solid solution of almandine and grossular, whose Raman spectra do not show any peak at ∼327 cm^−1^ (Fig. 1A–B). In addition, the analytical uncertainty of laser Raman analysis was about ± 0.8 cm^−1^ during our analytical session. The above arguments suggest that the peak at 327 cm^−1^ is unlikely caused by garnet. The peak at 354 cm^−1^, however, is indeed weak in quartz and can be influenced by garnet. Thus, its intensity may reflect a combined signal of quartz and garnet.

The fourth concern is the lack of other minor peaks, particularly that at 271 cm^−1^, which may help the identification of coesite. We have carefully re-checked the Raman spectra obtained during the same analytical session (Fig. 1). The peak at 271 cm^−1^ is indeed absent in the spectra. This can be caused by either the very tiny grain of the relict coesite or the direction of thin section which cuts off the oriented diffraction of coesite [6]. However, the peak at 174–178 cm^−1^ is weak but visible in the composite spectra, which is absent in individual garnet and quartz (Fig. 1). In addition, the peak at 151 cm^−1^, albeit very weak, is also present in the composite spectrum (Fig. 1G) but absent in those of garnet and quartz (Fig. 1). Both peaks, accompanied by the peak at 327 cm^−1^ and the most intense peak at 521 cm^−1^, are all representative for coesite. These observations have further reinforced the conclusion for the occurrence of coesite inclusions in the metamorphic garnet.

We agree with Compagnoni *et al.* [4] that the occurrence of coesite at the margin of quartz is not common, because coesite usually occurs in the center surrounded by quartz [1,2]. We have mentioned this important point in the paper of Chen *et al.* [3]. In the studied sample, we have observed many quartz inclusions in the garnet that are surrounded by radial cracks. It is this observation that inspired us to carry out numerous laser Raman analyses in order to find the occurrence of coesite in the garnet. Notably, in the UHP rocks of the Dora-Maira Massif, there are indeed examples showing that coesite occurs at the margin of quartz, i.e. between quartz and garnet, and one typical microphotograph was shown as Fig. S2 in the paper of Chen *et al.* [3]. Future work is needed to elucidate the conditions and implications for such an unusual occurrence of coesite in UHP metamorphic garnet. Notably, coesite also occurs as an intergranular phase along grain boundaries in the metamorphic rocks from the Dabie-Sulu orogen [7–9].

Compagnoni *et al.* [4] also mentioned that our proposed metamorphic evolution of the Eclogitic Micaschist Complex (EMC) in the Sesia zone is incorrect due to the inappropriate assumption of considering coesite stable with the peak mineral assemblage. However, as argued above, here we have clarified the concerns by Compagnoni *et al.* [4], and provided more compelling evidence for the presence of coesite in the Sesia zone. Thus, the proposed peak pressure should be above the quartz-coesite transition line.

In fact, previous studies have given a great variety of metamorphic P-T conditions and metamorphic ages for the rocks in the EMC of the Sesia zone [10–12], suggesting it may consist of multiple tectonic slices that have different P-T paths and histories of tectonic evolution. In our paper [3], we have highlighted the driving force for continental deep subduction in rifted continental margins. If the continental margin was attached to a wide and mature oceanic basin, continental deep subduction is usually attributed to gravitational pull of the previously subducting oceanic slab. In the Sesia zone, the oceanic basin could be very small so that its closure and subsequent continental deep subduction would be induced by distal push of either continental breakup or seafloor spreading [3]. Notably, this tectonic implication is also consistent with some previous results [13,14]. As such, our work has challenged the traditional paradigm of the driving force for continental deep subduction and thus opened a new window that can be explored by other approaches like numerical geodynamical modelling.

In summary, the above arguments may help to solve the concerns by Compagnoni *et al.* [4], because the Raman spectra provide the compelling evidence for the presence of coesite in the studied samples. Nevertheless, further work is needed to elucidate the following issues: (1) why the coesite occurs at the margin of quartz as inclusions in the garnet? (2) Are there any other localities or samples that contain coesite or other evidence of UHP metamorphism in the Sesia zone? (3) What is the geodynamic regime for the different P-T-t paths of multiple tectonic slices in the EMC unit of the Sesia zone? The answers to these questions require a series of comprehensive studies of petrology, geochronology and geodynamics for this interesting area in the future.
